# Rationale, design, and results of the first screening round of a comprehensive, register-based, *Chlamydia *screening implementation programme in the Netherlands

**DOI:** 10.1186/1471-2334-10-293

**Published:** 2010-10-07

**Authors:** Jan EAM van Bergen, Johannes SA Fennema, Ingrid VF van den Broek, Elfi EHG Brouwers, Eva M de Feijter, Christian JPA Hoebe, Rik H Koekenbier, Eline LM Op de Coul, Sander M van Ravesteijn, Hannelore M Götz

**Affiliations:** 1 STI AIDS Netherlands, Amsterdam, The Netherlands; 2 Amsterdam Public Health Service, Amsterdam, The Netherlands; 3 Rotterdam Rijnmond Public Health Service, Rotterdam, The Netherlands; 4 Department of Infectious Diseases, South Limburg Public Health Service, Geleen, The Netherlands; 5 Centre for Infectious Disease Control, National Institute of Public Health and the Environment, Bilthoven, The Netherlands

## Abstract

**Background:**

Implementing *Chlamydia trachomatis *screening in the Netherlands has been a point of debate for several years. The National Health Council advised against implementing nationwide screening until additional data collected from a pilot project in 2003 suggested that screening by risk profiles could be effective. A continuous increase in infections recorded in the national surveillance database affirmed the need for a more active approach. Here, we describe the rationale, design, and implementation of a *Chlamydia *screening demonstration programme.

**Methods:**

A systematic, selective, internet-based *Chlamydia *screening programme started in April 2008. Letters are sent annually to all 16 to 29-year-old residents of Amsterdam, Rotterdam, and selected municipalities of South Limburg. The letters invite sexually active persons to login to http://www.chlamydiatest.nl with a personal code and to request a test kit. In the lower prevalence area of South Limburg, test kits can only be requested if the internet-based risk assessment exceeds a predefined value.

**Results:**

We sent invitations to 261,025 people in the first round. One-fifth of the invitees requested a test kit, of whom 80% sent in a sample for testing. The overall positivity rate was 4.2%.

**Conclusions:**

This programme advances *Chlamydia *control activities in the Netherlands. Insight into the feasibility, effectiveness, cost-effectiveness, and impact of this large-scale screening programme will determine whether the programme will be implemented nationally.

## Background

Screening for *Chlamydia trachomatis *aims at early detection and treatment of asymptomatic infections, which limit the spread of infection in the population and reduce complications in infected individuals [1,2].

A large population-based study in four regions of the Netherlands in 2003 (the *C. trachomatis *pilot study) showed an overall prevalence of 2.1% of C. trachomatis among participants aged 15-29 years, with positivity rates in urban areas five times as high as those in rural areas (3.2% vs. 0.6%) [3]. In this study, positivity correlated closely with several risk factors such as increasing numbers of sex partners, female gender, and specific ethnic background (up to 8% among Surinamese and Dutch Antillean participants). Outcomes of this study were used to develop a risk score that allowed a more selective - and therefore more cost-effective - screening model [4].

In 2006, the National Health Council, considering the substantial increase in *C. trachomatis *infections, reconsidered its reluctant position against screening, and favoured developing a demonstration project to increase the level of *C. trachomatis *testing in the Netherlands and to supplement the then current *C. trachomatis *testing, which was mainly provided by general practitioners (GPs) and sexually transmitted infection (STI) clinics [5].

In this paper, we describe the rationale, design, and roll-out of a large-scale register-based, systematic, selective screening programme, in which the internet plays a major role. The participation criteria differ in rural and urban areas. We also give a brief outline of process and impact evaluation studies that accompany the screening, and we present the results of the first screening round.

## Methods

### Design of the screening programme

STI AIDS Netherlands coordinates the screening programme, and the local Public Health Services carry it out, expanding the screening in a phased implementation (stepped wedge, cluster, randomised design). The Centre for Infectious Disease Control, a section of the National Institute for Public Health and the Environment (RIVM), is responsible for the formal evaluation process. An advisory committee consisting of experts and other relevant parties, including the Ministry of Health, support the executive board of the screening programme. The Ethics Committee of the Free University of Amsterdam (METC number: 2007/239) has approved the study, which conforms to national and international legislation. Participants provide online informed consent. The time frame of the demonstration project is 2008-2010.

### Rationale for register-based screening

We chose to organise the screening as register-based (i.e. all individuals registered in the target population are actively approached for participation) as opposed to opportunistic (screening offered during consultations or outreach).

The only two published randomised controlled trials (RCTs) investigating the individual benefits of screening assessed systematic screening [6,7] and have shown a reduction in pelvic inflammatory disease (PID). A new trial has recently been added to this body of evidence [8]. The programme coverage (or effective test rate) is critical for achieving not only individual benefit (reduction of complications by means of early diagnosis and treatment), but also a reduction of transmission at the population level. The reported population coverage of ongoing opportunistic programmes is limited [9]. In contrast, a Danish study has shown that the eligible population was 11 times as likely to be tested in home-based screening as in the opportunistic testing that doctors offer during routine care [10]. In addition, a recent review shows that the evidence base for opportunistic screening is poor [11,12]. We have demonstrated the feasibility and acceptability of register-based systematic screening in the Netherlands via the Municipal Health Services in a previous study [3,13].

### Rationale for population selection for screening

The demonstration programme has been implemented in three regions: two large cities (Amsterdam and Rotterdam) and one somewhat more rural area (South Limburg). Each year, all residents aged 16-29 years receive an invitation letter. Our previous research showed that prevalence in the population of highly urbanised areas in the Netherlands is well above 3% [3]. People living in Amsterdam and Rotterdam are advised to participate if they are (or have been) sexually active. In areas of lower population density, the population prevalence is between 0.6% and 2%, so that selective screening based on risk profiles is required to reach a better cost-effectiveness profile [3,4]. Therefore, eligibility for screening in South Limburg is assessed on the basis of individual risk scores calculated from invitees' information online. The web-based questionnaire asks nine questions about demographics and sexual behaviour, and each item contributes to the risk score (Additional file 1). The invitee can request a test kit only if the risk score reaches a predefined value. The prediction rule for this selective screening is based on previous research [4] and aims at a *C. trachomatis *positivity yield of 4-5%.

We chose to include both men and women in the screening. Population studies show prevalence rates among men comparable to those among women. Including men in the screening makes them part of the solution rather than the problem [14]. Models show that screening both men and women can have more impact than screening only women [15]. However, the impact of screening both men and women on community prevalence has yet to be demonstrated in real life. In fact, a recent literature review called for more high-quality RCTs to ascertain the impact on individual complications like PID and infertility, as well as the impact on community prevalence [12]. We are expanding our screening area by area, and we are including *all *eligible residents of a geographically confined area within a limited time. This strategy covers social and sexual networks in order to grasp any community impact of accelerated screening.

### Logistics

**Figure 1:**
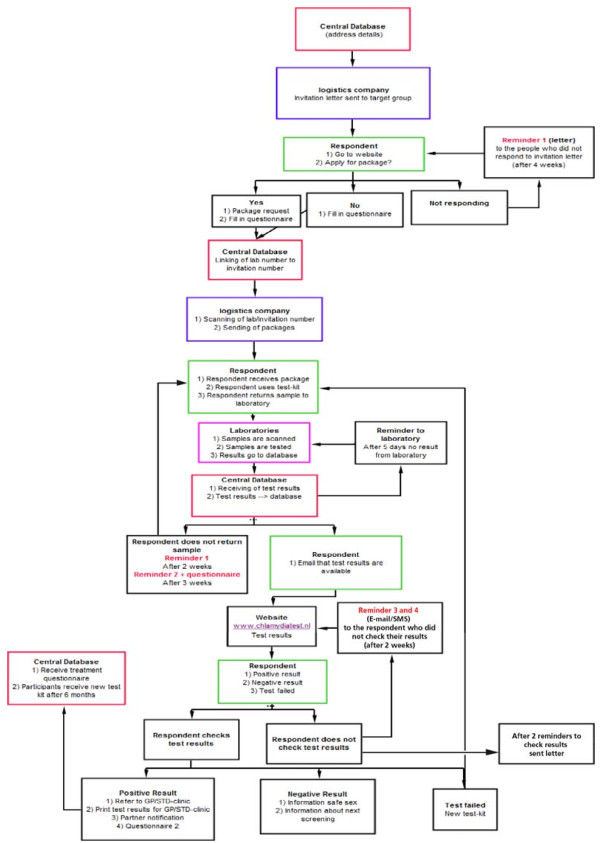
**Operational flowchart of the *Chlamydia *screening programme**.

**Figure 2:**
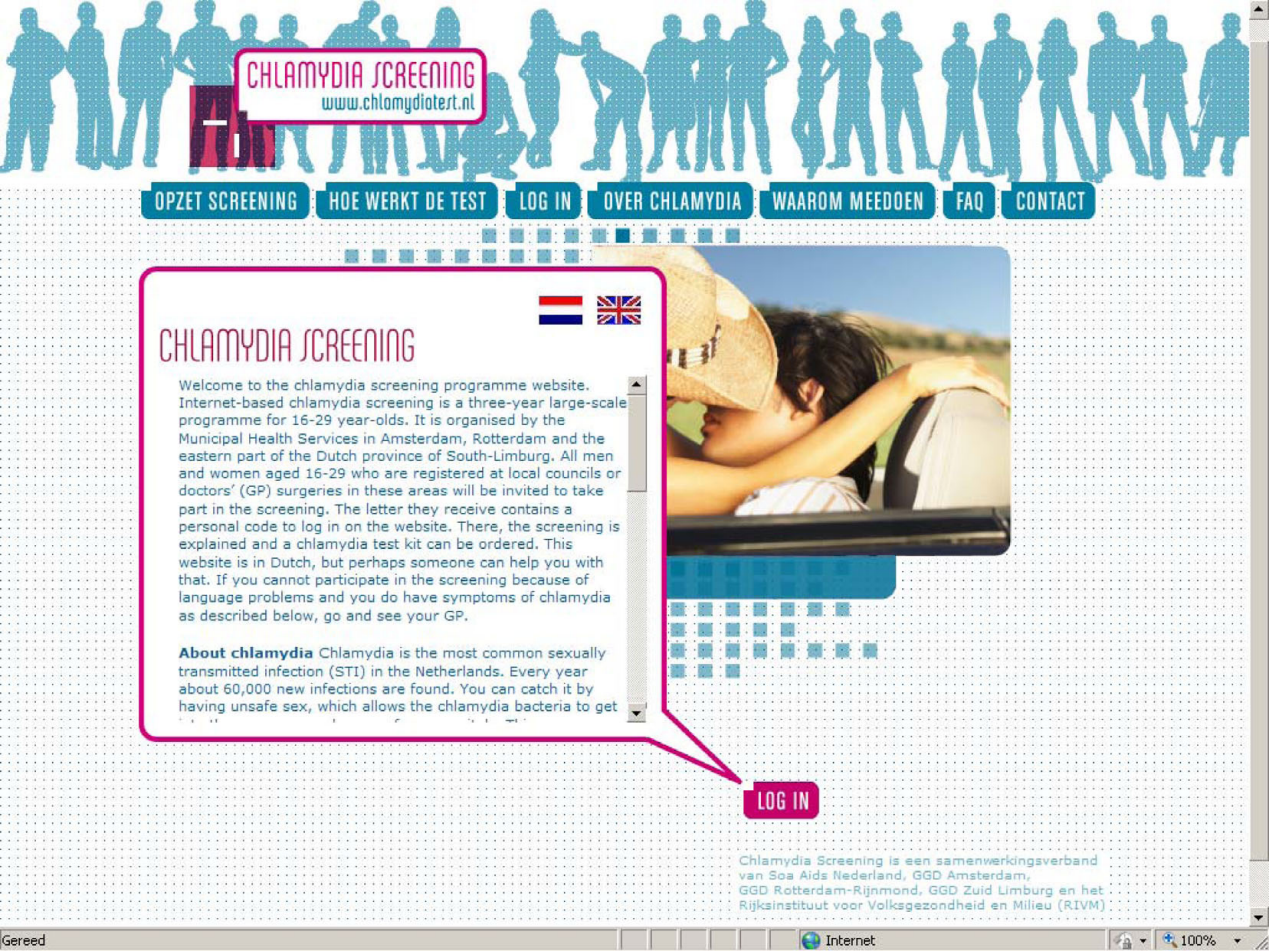
**Website of the *Chlamydia *screening programme**.

Figure 1 presents the framework of the logistics. The local Public Health Services send invitations to all 16 to 29-year-olds in Amsterdam, Rotterdam, and South Limburg. Municipal population registers supply the addresses. The order of invitations is cluster-randomised per area or municipality according to postal codes lists within clusters in each of the three participating regions. The invitations contain an information leaflet and a letter, advising those who are (or have been) sexually active to participate. The invitation letters provide a unique ID number (login code) that enables the invitee to request a test kit at http://www.chlamydiatest.nl (Figure 2). The invitation letter and information materials (text and website) have been developed and pretested with the relevant age, sex, and cultural groups. In the lower prevalence area, South Limburg, anyone who visits the website must first do a short online risk assessment, and can only request a test kit if the risk score reaches a predefined level of 6 or more, compatible with an estimated positivity rate of 4-5% (see Additional file 1).

Those who have not reacted to the initial invitation within 4 weeks receive a reminder invitation. A reminder is e-mailed to those who do not send a sample to the laboratory within 2 weeks of their request for a test kit; a second reminder is e-mailed 1 week later. Invitees who send a sample to the laboratory receive an e-mail and/or SMS to inform them when their test result is available online. *Chlamydia trachomatis *positive participants can print the PDF letter with their test results and information for health care providers. The test results on the website can only be accessed by personal login. If the test results are not accessed within 14 days, a reminder e-mail is sent. Everyone with a positive result who has not checked the test result within 4 weeks is sent a letter with the result at the address provided.

**Figure 3:**
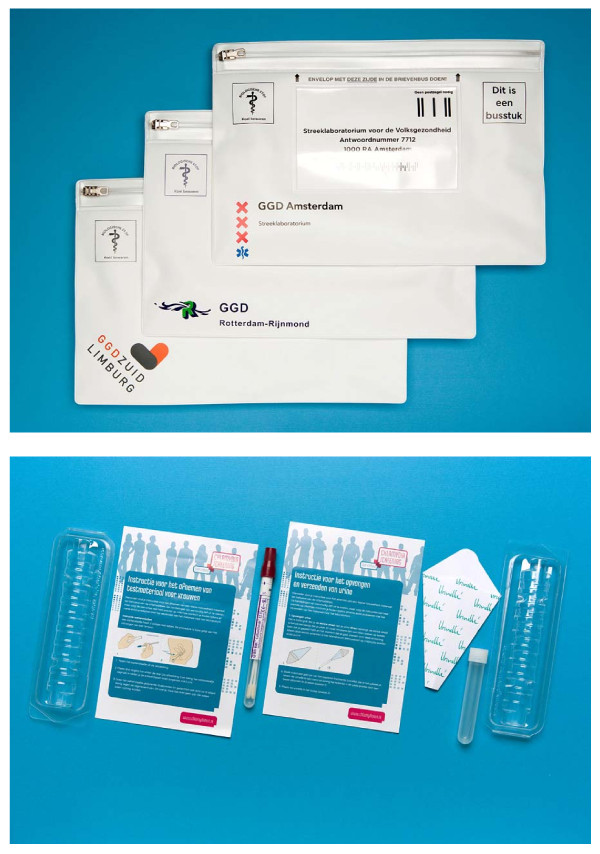
**Polymed plastic envelope and test materials**.

The test kit includes a home sample kit (Figure 3; men: urine sample, women: vaginal swab or urine sample). The participants send the self-collected samples to the regional laboratory in an envelope suitable for mailing biological materials. The three participating laboratories are all certified, take part in quality control programmes and use certified nucleic acid amplification techniques according to the manufacturer's guidelines. The one laboratory that uses pooling retests positive pools individually. The two laboratories that do not pool confirm positive tests with a second test on the same specimen.

The patient's GP provide treatment, but if a participant prefers, the local STI centre provides it. The current sex partners of participants with positive test results are advised to be treated simultaneously or seek prompt testing and treatment. Previous partners are notified predominantly by patient referral. Additionally, participants with positive test results can also use the website, anonymously if they wish, to notify previous sex partners of the last 6 months via e-mail. These partners are offered testing within the existing screening programme. Participants who test positive receive a new test kit automatically after 6 months.

### Data collection and evaluation

The programme's central database automatically stores data from all invitees. The database includes data pertaining to progression through the screening process and data from the local population registers, the laboratories, and the company responsible for handling the mailings. The central database enables process monitoring, evaluation, and uploading of regular time-framed overviews of key outcomes and indicators of programme performance. Data obtained from online questionnaires for participants and mailed questionnaires for non-responders, including characteristics such as self-reported sexual history and clinical symptoms, are also linked to the central database to allow for more detailed analyses.

The evaluation of the screening implementation consists of various components. A detailed description of the design of the evaluation is given elsewhere [16]. The first part concerns process evaluation, including non-response and acceptability studies [17]. In the second part, the effects of screening are evaluated. For evaluation purposes, we chose a phased implementation of the screening. This 'stepped-wedge design' enables a sequential roll-out in geographical clusters (neighbourhoods), randomised within a number of periods. The design allows for:

1. Estimation of the effect of one or two screening rounds on the population prevalence of *C. trachomatis *and self-reported PID.

2. Determination of trends of participation in different screening rounds.

3. Assessment of baseline ecological trends among those who have not received a screening invitation at the end of the second screening round.

Other important components of the evaluation are the modelling of positivity and prevalence, and the cost-effectiveness analyses.

## Results

### The first screening round

Between April 2008 and February 2009, 261,025 people were sent an initial invitation for the first screening round (140,058 in Amsterdam, 107,806 in Rotterdam, and 13,161 in South Limburg). Overall, 21% of those invited were 16-20 years old, 37% were 20-24 years old, and 42% were 25-29 years old; 52% of the invitees were female. One percent of the invitational letters were returned as undeliverable.

### Request rate

The request rate - i.e. the proportion of the invited population requesting a test kit - was 11.9% within 4 weeks after the initial invitation. The remaining invitees who did not react within 4 weeks received a reminder invitation, after which an additional 8.3% requested a test kit. In total, 52,741 people requested test kits upon invitation. The overall request rates were 20.5% in both urban areas compared with 13.8% in sub-urban South Limburg. In this region, eligibility for a test kit was dependent on a sufficiently high risk score. Of the 22.5% of the invitees who filled in the online risk score, 63% were eligible. In all three regions, the request rates were substantially higher among women than among men (25.8% vs. 14.1%, χ^2 ^*p *< 0.0001). They were also higher among those aged over 20 years than among younger groups (22% vs. 13.4%, χ^2 ^*p *< 0.0001; Table 1).

**Table 1:** Proportions of the invited population requesting test kits in the *Chlamydia *screening implementation, 2008-2009

	Amsterdam			Rotterdam			South Limburg*			All regions		
	
	Men	Women	Total	Men	Women	Total	Men	Women	Total	Men	Women	Total
*n*	65268	74790	140058	52692	55114	107806	6848	6313	13161	124808	136217	261025
Age group (years)	Proportions by region, gender, and age groups (in percentages)											
16-19	9.1	18.4	13.9	8.9	17.6	13.2	5.2	17.7	11.3	8.8	18.0	13.4
20-24	14.8	27.9	22.0	14.2	26.1	20.3	9.2	21.7	15.1	14.2	26.9	21.0
25-29	17.7	30.4	24.5	16.3	26.9	21.7	10.0	19.1	14.4	16.8	28.6	23.0

All age groups	15.0	27.2	21.5	13.8	24.5	19.3	8.4	19.6	13.8	14.1	25.8	20.2

*In this region, 22.5% of the total invited population completed the online risk score, and 63% of the scores were high enough for a test kit request.

**Figure 4:**
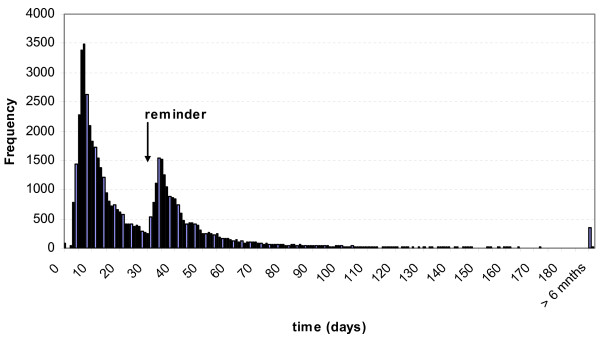
**Time between invitation and request of a testing kit**.

The median time lapse between the invitation and request for a test kit was 18 days (range 0-428 days). On average, people requested a test kit 28 days after the invitation [standard deviation (SD) ± 30 days]. Reminders significantly increased the requests for a test kit: 33% of the requests for test kits were received after the reminder was sent (Figure 4).

### Effective participation rate

Of those who requested a test kit, 41,637 (79%) sent in a sample. This test uptake of 79% translates into a programme coverage (effective participation or test rate) of 16% of the invited population being tested in the first screening round.

The participation rate was considerably higher among women (21.0%) than among men (10.4%, *p *< 0.0001), and among those 20 years old and older (17.6%) than among those less than 20 years old (9.9%, *p *< 0.0001). The trends were similar across regions, but there were significant differences in overall participation rates between regions (Table 2).

**Table 2:** Proportions of the invited population who provided samples in the *Chlamydia *screening implementation, 2008-2009

	Amsterdam			Rotterdam			South Limburg*			All regions		
	
	Men	Women	Total	Men	Women	Total	Men	Women	Total	Men	Women	Total
*n*	65268	74790	140058	52692	55114	107806	6848	6313	13161	124808	136217	261025
Age group (years)	Proportions by region, gender, and age groups (in percentages)											
16-19	5.9	14.1	10.1	6.3	13.5	9.9	3.9	14.4	9.0	6.0	13.8	9.9
20-24	10.4	23.1	17.4	10.9	20.9	16.1	6.5	18.1	12.0	10.4	22.0	16.6
25-29	13.3	25.4	19.8	12.6	21.8	17.3	7.8	15.9	11.7	12.8	23.6	18.5

All age groups	10.8	22.4	17.0	10.5	19.6	15.2	6.2	16.2	11.0	10.4	21.0	16.0

*In this region, 22.5% of the total invited population completed the online risk score, and 63% of the scores were high enough for a test kit request

The median time lapse between requesting a test kit and returning a sample to the laboratory was 11 days (range 0-390; mean 17 days, SD ± 19). Reminders significantly contributed to the sending of samples: 39% of samples were received after the reminder invitation was sent. See Op de Coul et al. [17] for more detailed and time-framed monitoring indicators of process performance.

### Positivity for Chlamydia trachomatis

The laboratories processed 96% of the samples within the predefined target of 10 working days, and test results were made available online. A total of 1758 people tested positive for *C. trachomatis*, which resulted in an overall positivity rate of 4.2% (95% CI 4.0-4.4%).

In this first screening round, the positivity rate was significantly lower among men [3.8% (95% CI 3.5-4.15%)] than among women [4.4% (95% CI 4.2-4.7%), *p *= 0.003]. Those younger than 20 years were significantly more likely to have positive test results (7.3%) than those 20 years old or older (3.8%, *p *< 0.0001). In particular, younger girls were more likely to test positive (8%). We also found substantial differences in the positivity rates across the participating regions (Table 3).

**Table 3:** Proportions of positive samples for the various groups in the *Chlamydia *screening implementation, 2008-2009

	Amsterdam			Rotterdam			South Limburg*			All regions		
	
	Men	Women	Total	Men	Women	Total	Men	Women	Total	Men	Women	Total
*n*	7050	16777	23827	5544	10817	16361	426	1024	1450	13020	28618	41638
Age group (in years)	Positive samples per group (in percentages)											
16-19	5.2	6.9	6.4	6.3	9.9	8.7	5.2	4.5	4.7	5.7	8.0	7.3
20-24	3.9	4.3	4.2	4.4	5.9	5.4	5.6	5.9	5.8	4.1	5.0	4.7
25-29	2.6	2.5	2.5	3.7	3.6	3.6	5.4	4.3	4.7	3.1	2.9	3.0

All age groups	3.3	3.7	3.6	4.3	5.5	5.1	5.4	5.0	5.1	3.8	4.4	4.2

*In this region, 22.5% of the total invited population completed the online risk score, and 63% of the scores were high enough for a test kit request

### Treatment

Ninety-five percent of the participants checked their test results online, and 90% did so within 7 days after being notified by e-mail that the results were available online. Preliminary analyses of the questionnaires, filled in by 43% of all those who were *C. trachomatis *positive, indicated that an estimated 91% of them saw a physician (82% went to their GP). Of those who consulted a physician, 98% received treatment. Patient referral is the mode of tracing contacts in the Netherlands, but anonymous, web-based, partner notification via the programme was also possible. The patients with *C. trachomatis *provided e-mail addresses for notification of 382 non-regular sex partners of the previous 6 months. Altogether, 125 of the notified partners (33%) requested a test kit, of whom 107 (86%) sent in a sample for *C. trachomatis *testing. Twenty-nine of these samples (27%) tested positive.

Participants who were *C. trachomatis *positive were automatically sent a new test kit at the address provided 6 months after the initial test. Of these participants, 1152 (68%) sent in a lab sample, and 95 *C. trachomatis *test results were positive - a positivity rate of 8.2% (95% CI 6.6-9.7%).

Acceptability and usability studies are currently in progress among both participants and non-responders. Preliminary data indicate overall good acceptability, with participants showing positive attitudes towards the screening programme and communication via the website and by SMS. In our postal non-response study, we did not find indications that participation in the screening was hampered by limited access to the internet. Further detailed analyses regarding acceptability, process, and non-response will generate more in-depth insight into factors associated with participation, and coverage. These analyses will be reported separately.

## Discussion

After taking the best available evidence into account and after the pilot study provided insight into the population prevalence of *C. trachomatis *in urban and rural areas, we embarked on a register-based, systematic, selective, internet-based programme for the *Chlamydia *screening implementation (CSI). The entire population aged 16-29 years in geographically defined areas is annually invited for screening to include any community effect of repeated screening rounds on prevalence. Only sexual active people are advised to participate, and people residing in lower prevalence areas must take part in an online risk assessment in order to request a test kit. The screening is being rolled out in randomised clusters in a stepped-wedge design to enable impact evaluation.

In the first screening round, the 52,741 people who requested a test kit represented 20% of the invitees. Almost 80% of those with a request sent in a sample, so that the overall participation rate was 16%. Participation among women (21%) was double that among men (10%). This overall participation rate is lower than the rate in the pilot study (30%) [3]. Differences in participation may be partially explained by differences in offering the testing kit. In the pilot study, test kits were sent directly with the invitation instead of having the test kits requested via internet. An advantage of our current approach was lower cost: most test kits are not used if sent to every eligible person. A study in Denmark has also found that direct mailing compared to requesting a kit for home-based testing lowered the participation rate a little for women, but substantially for men [10].

Another factor that contributed to the lower participation rate is the selective nature of our screening: only sexually active people are eligible, and in the lower prevalence area of South Limburg, only sexually active people with a risk score of a predefined value may participate. For example, in the group of 16-year-olds, only an estimated 40% can consider participation because the rest are not yet sexually active [18]. Nonetheless, the overall effective test rate of 16% seems low and makes it even more important to know who is participating and who is not. Our acceptability and non-response studies will attempt to determine whether participation is biased toward the 'worried well' and whether non-responders make valid 'informed' decisions not to participate if they perceive themselves at lower risk. Moreover, our future studies will have to show if this systematic screening, which is additional to the existing testing in health facilities, can reduce community prevalence.

We are cautious about international comparisons of screening coverage because programme designs may differ and denominators may not be comparable. We use the total population of the relevant age group as the denominator. Sometimes denominators for coverage include only sexually active people or - as in opportunistic programmes - the people who accepted the offer of a test or the attendees at health facilities.

*Chlamydia trachomatis *positivity among the participants in the screening programme is 4.2%. We encountered substantial differences within and between the two large cities, and we are currently analysing community risk profiles. In the lower-prevalence area of South Limburg, selection by risk score on the internet worked well and increased positivity from 1.3% in the pilot study population to 5.1% in the current screening programme.

The positivity rate for the 25 to 29-year-olds is 2.9%, which is substantially lower than that among younger people. Cost-effectivity analyses will have to show whether this age group should be included for universal screening in very urbanised areas, or whether this group needs the more selective screening that we do in less urbanised areas.

Compared to the 16% overall participation rate, a reassuring finding was that 68% of those who were *C. trachomatis *positive did participate when they automatically received a re-screening invitation 6 months later. As others have reported [19,20], the re-infection rate was high (8.2%). Reported treatment rates are well above 90%, and they are in line with the well-documented treatment rates in our pilot study [21]. These facts pinpoint the need for early regular testing of this group, as well as for more effective patient management, including counselling and partner management [22,23].

The use of internet, SMS, and e-mail services made communication flexible and 'future proof' for the next young generations. These services provided new opportunities for reminders and partner notification. The development of computer software and the project's database enable easy adaptation of the screening in time and expansion to other geographic areas in the future.

## Conclusions

We have reported the rationale, design, and results of the first screening round of our selective, systematic, internet-based programme for *C. trachomatis *screening. We designed the programme to show the impact on issues such as population prevalence and self-reported PID, thereby contributing to the body of evidence for or against screening. More evidence of the impact and cost-effectivity of real-life comprehensive programmes for *C. trachomatis *screening is urgently needed. Currently, much of the evidence in favour of screening derives from selected studies, the strengths of which have been questioned [12]. Most studies involve a follow-up of only one screening round and focus on prevention of PID as an end-point, which is only one of the goals of screening. Our programme is now entering the next phase: we are implementing the second screening round. This will help us gain more evidence about the sustainability of a screening programme and the rates of participation and positivity in successive screening rounds. The outcome of this 3-year demonstration programme will be crucial for the decision about a national roll-out of *C. trachomat*is screening in the Netherlands.

## Competing interests

The authors declare that they have no competing interests. The *Chlamydia *Screening Implementation Programme is being carried out by request of the Ministry of Health, Welfare and Sport. The Dutch Organisation for Health Research and Development (ZonMw; project number 12.400.0001) is funding the project.

## Authors' contributions

All authors are part of the CSI Project Group, which is involved in the conception, design, and implementation of the *Chlamydia *Screening Implementation Programme. JvB and EF were responsible for the coordination of the screening in the three regions; CH and EB, for the screening in South Limburg, JF and RK in Amsterdam, and HG and SR in Rotterdam. JvB and JF drafted the manuscript. Other authors provided feedback for the initial document and contributed to the revision of the paper. All authors read and approved the final draft.

## Pre-publication history

The pre-publication history for this paper can be accessed here:

http://www.biomedcentral.com/1471-2334/10/293/prepub

## Supplementary Material

Additional file 1 **Calculation of risk score in the low prevalence area**. Click here for file
