# Supplementary material for: Rationale, design, and results of the first screening round of a comprehensive, register-based, Chlamydia screening implementation programme in the Netherlands
Source: BMC Infect Dis. 2010 Oct 7;10:293. doi: 10.1186/1471-2334-10-293 (PMC2959064; doi:10.1186/1471-2334-10-293)
Supplement: Additional file 1 — Calculation of risk score in the low prevalence area. [file 1471-2334-10-293-S1.DOC]

| **Question** | **Answer** | **Points added to score** | |
| --- | --- | --- | --- |
| Age | 15–19 years | 1 |  |
|  | 20–29 years | 0 |  |
| Place of residence | Area not or hardly urbanised | 0 |  |
|  | Area somewhat urbanised | 2 |  |
| Level of education | Higher | 0 |  |
|  | Intermediate or basic | 2 |  |
| Ethnicity | Dutch | 0 |  |
|  | Antillean or Surinamese | 2 |  |
|  | Other | 0 |  |
| Recent blood loss after sexual intercourse | Yes | Women: 1 | Men: 0 |
|  | No | 0 |  |
| Recent increased urge to urinate | Yes | Men: 2 | Women: 0 |
|  | No | 0 |  |
| Condom used at last sexual intercourse | Yes | 0 |  |
|  | No | 1 |  |
| Lifetime sex partners | 1 partner | 0 |  |
|  | 2–5 partners | Women: 3 | Men: 2 |
|  | 6 or more partners | Women: 5 | Men: 3 |
| New sexual partner in the last 6 months | Yes | 1 |  |
|  | No | 0 |  |
